# Gestational age specific anthropometric postnatal percentile charts for neonates born at tertiary hospital in Eastern Nepal

**DOI:** 10.1186/s12887-022-03157-w

**Published:** 2022-02-15

**Authors:** Anjum Shakya, Nisha Keshary Bhatta, Rupa Rajbhandari Singh, Shankar Prasad Yadav, Jitendra Thakur

**Affiliations:** 1 Department of Pediatrics, B.P.Koirala Institute of Health Sciences, Dharan, Nepal; 2 Division of Neonatology, Department of Pediatrics and Adolescent Medicine, BPKIHS, Dharan, Nepal; 3 Department of Pediatrics and Adolescent Medicine, BPKIHS, Dharan, Nepal

**Keywords:** Percentile charts, Birth weight, Head circumference, Length, Nepal

## Abstract

**Background and objectives:**

Birth weight, Head circumference (HC), and Length are important clinical indicators for evaluation of prenatal growth and identification of neonates requiring detail assessment and monitoring. Gestational age-specific percentile charts are essential tool for both obstetricians and pediatricians in their day to day practice. This study aimed to develop gestational age specific percentile chart of Birth weight, Length and HC for neonates.

**Methods:**

In this Cross sectional observational study, HC, Birth weight and Length of live singleton neonates from 28 to 42 weeks of gestation fulfilling the inclusion criteria were measured over a period of one year. Mean, standard deviation, and percentiles values for different gestational age were calculated. Graphs were constructed using two way graph and Lowess smoothening method.

**Results:**

Of total 2662 neonates, male: female ratio was 1.3:1 with maximum neonates in 40 weeks of gestation. The mean Birth weight, HC and Length was 2852.02 gm, 33.6 and 48.42 cm respectively. Overall males have more mean weight than females by 46.35gms. However, mean HC of male and female were similar 33.6 and 33.61 cm respectively and on average males were 0.27 cm longer compared to female. The mean Birth weight, HC and Length at 40 weeks was 3123.43gm (± 427.82), 34.249 cm (± 0.87) and 49.61 cm(± 1.85) respectively. The 10^th^, 50^th^ and 90^th^ percentile at 40 weeks for Birth weight being 2550gm, 3100gm and 3750gm respectively. The gestational age specific percentile chart and growth curve are appropriately placed in the manuscript.

**Conclusions:**

The percentile charts in this study may be used as reference for local population and similar data from various parts of the nation can provide a national reference curve for healthy neonates.

## Background

The neonatal anthropometric indicators - birth weight, length and head circumference(HC) are of utmost importance for evaluation of prenatal growth and identification of infants that require thorough assessment and close monitoring postnatally.

Birth weight is also a valuable indicator of maternal health, nutrition and quality of antenatal services, and for the monitoring of epidemiological outcomes and public health care policies [1, 2]. Neonatal outcome of babies with different gestation varies despite of similar weight [3].

Similarly, long infants are at higher risk of perinatal mortality, and excessive variation in HC can denote malformation of the central nervous system secondary to genetic or chromosomal abnormalities or teratogenic insults [4, 5].

Based on ethnically mixed population in Colorado, the first gestational age specific anthropometric reference chart was developed by Lubchenco, et al. in 1963, which is still used in many centers [6]. These centile reference charts are used to monitor clinical measurements on individuals in the context of population values [7]. However, these charts are not universally applicable because the growth potential of the fetus is influenced by various factors including sex of the infant, ethnic group and geographical factors [8]. Changes in the parity, socioeconomic and environmental conditions necessitate an update in the existing growth charts.

Similar growth charts have been published by various authors representing growth patterns of diverse population in different parts of the world. The objective of present study is to construct percentile charts for birth weight, length and head circumference for infants born from 28 to 42 weeks of gestation to suit the neonatal population of Nepal.

## Method

This study was a cross-sectional observational study conducted over a period of one year (August 2015 to July 2016) at a tertiary center, Bishweshwar Prasad Koirala Institute of Health Sciences (BPKIHS), Dharan, Nepal. All the singleton live birth from 28 to 42 weeks of gestation within 24 h of birth, delivered during the study period were included in the study by consecutive non- purposive sampling method. The ethical approval was taken from the Institutional Review Committee (IRC) of BP Koirala Institute of Health Sciences.

Gestational age was estimated by first day of the last menstrual period (LMP). In cases where LMP was unknown or in clinically discrepant cases, it was confirmed by clinical assessment using New Ballard’s scoring system or first trimester ultrasonography (USG). The gestational age estimated by scoring was included if the difference between LMP and scoring was more than two weeks.

Birth weight was measured within 24 h of birth after drying without clothing on the electronic weighing machine EBSA-20 to the nearest ±5 g with maximum up to 20 kg and calibrated before each measurement.

Length was recorded placing the child supine to the nearest 0.1 cm using infantometer. The head was held firmly in position against a fixed upright head board by one person. Legs were kept straight, keeping feet at right angles brought into firm contact with the child’s heels.

Head Circumference was measured at 24 to 48 h of life with the locally available non-stretchable measuring tape, the maximum circumference of the head from the occipital protuberance to the supraorbital ridges on the forehead to the nearest 0.1 cm was recorded.

Name of mother, birth weight, length and HC of enrolled newborn were recorded. Multiple birth, gross congenital malformations and hydrops, still birth, maternal complications (e.g. Pre-eclampsia/ eclampsia, Gestational Diabetes Mellitus (GDM), severe anemia, maternal medical disorders.), large caput succedaneum and cephalhaematoma were excluded.

Data entry was done in Microsoft 2007 Excel. Data analysis was performed in Microsoft 2007 Excel and Stata IC 14.1. The percentiles (3^rd^, 10^th^, 25^th^, 50^th^, 75^th^, 90^th^, 95^th^, and 97^th^ ) for the birth weight, length and HC according to gestational age was calculated, and charts constructed using Stata IC 14.1 Growth curves were constructed using two way graph and Lowess smoothening method.

## Results

**Fig. 1:**
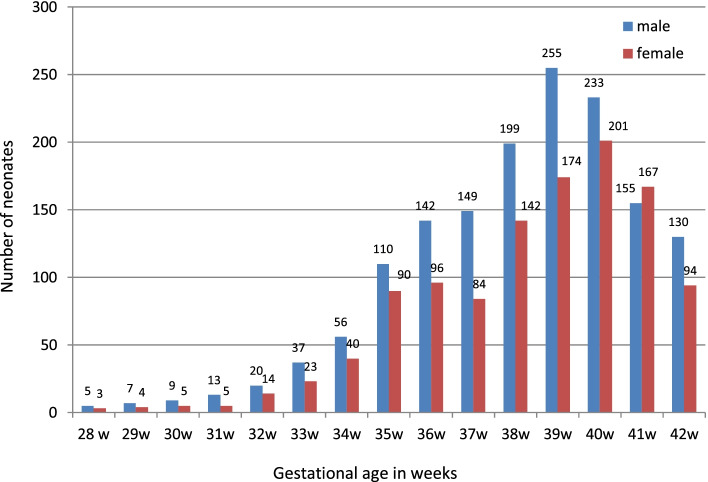
Gender wise distribution of neonates according to gestational age

Out of 2662 live singleton normal newborns included in the study, 1520 were male and 1142 were female with male: female ratio of 1.3:1. The maximum neonates (n) were in 40 weeks of gestation (Fig. 1).

The mean birth weight for this study population was 2852.02 gm (±571.89). Mean birth weight of male was 2871.9gm (±600.78) and of female was 2825.55gm (±530.13). Similarly, mean length of male newborn was 48.52 cm (±2.64) and of female newborn was 48.25 cm (±2.25). On an average male newborn length was 0.27 cm more than female newborn. The mean head circumference of the newborn was 33.6 cm and both male and female newborn had the similar mean head circumference.

According to gestational age of newborn, the mean ± SD of birth weight, head circumference and length were calculated (Table 1).

**Table 1:** Mean birth weight, head circumference and length of neonates according to gestational age

Gestational age (in weeks)	Mean weight ± SD (in grams)	Mean HC ± SD (in centimeter)	Mean length ±SD (in centimeter)
28	1131.25 ± 186.96	26.75 ± 1.41	36.98 ± 3.81
29	1563.64 ± 423.14	29.59 ± 2.49	43.65 ± 2.47
30	1535.71 ± 332.49	29.16 ± 3.87	42.49 ± 3.87
31	1561.11 ± 325.2	29.58 ± 1.71	43.88 ± 1.8
32	1717.94 ± 365.91	29.26 ± 1.76	43.99 ± 2.97
33	1848.33 ± 377.32	30.66 ± 1.54	44.21 ± 2.16
34	2251.04 ± 438.96	32.03 ± 1.73	45.95 ± 1.74
35	2328.23 ± 373.77	32.6 ± 1.71	46.42 ± 1.49
36	2594.45 ± 445.56	33.19 ± 1.21	47.24 ± 1.69
37	2730.9 ± 427.62	33.48 ± 1.17	47.9 ± 1.86
38	2971.03 ± 396.99	33.92 ± 1.03	49.09 ± 1.86
39	3057.76 ± 403.05	34.133 ± 0.9	49.29 ± 1.79
40	3123.43 ± 427.82	34.249 ± 0.87	49.61 ± 1.85
41	3193.39 ± 398.09	34.406 ± 0.83	49.85 ± 1.76
42	3136.83 ± 442.43	34.328 ± 0.72	49.22 ± 1.72

The 50^th^ percentile for birth weight, HC and length for neonates at 40 weeks of gestation were 3100gm, 34.2 and 49.8 cm respectively. The gestational age specific percentile of different anthropometric variables are shown in Tables 2, 3 and 4.

**Table 2:** Percentile values of birth weight (grams) for each gestation from 28 to 42 weeks

Gestational age (in weeks)	3^rd^	10^th^	25^th^	50^th^	75^th^	90^th^	97^th^
**28**	950	950	950	1125	1287	1330	1379
**29**	1150	1160	1250	1400	2000	2050	2330
**30**	1000	1100	1250	1500	1825	2025	2030.
**31**	1200	1245	1300	1500	1712.	2205	2224.
**32**	1231	1275	1500	1675	1825	2250	2795
**33**	1207.	1500	1612.	1800	2000	2195	2877.
**34**	1432	1720	2000	2250	2400	2800	3500
**35**	1500	1910	2162.	2350	2500	2745	3248.
**36**	1755.	2000	2300	2600	2850	3205	3500
**37**	2000	2100	2500	2750	3000	3280	3500
**38**	2250	2500	2700	3000	3250	3500	3750
**39**	2445	2500	2750	3000	3250	3600	3900
**40**	2300	2550	2800	3100	3462	3750	3947.
**41**	2500	2750	2900	3200	3450	3750	4065.
**42**	2300	2500	3862.	3150	3300	3750	4012.

**Table 3:** Percentile values of head circumference (centimeter) for each gestation from 28 to 42 weeks

Gestational age (in weeks)	3^rd^	10^th^	25^th^	50^th^	75^th^	90^th^	97^th^
**28**	25.7	25.7	25.9	26.5	28.6	28.6	28.8
**29**	26.4	26.5	27	29.8	32	32.8	33
**30**	25.5	26	27.5	28.6	31.1	32.1	32.6
**31**	27	27	28.2	29.4	30.8	32.1	32.5
**32**	27	27	27.9	29	30.5	32.1	32.7
**33**	27	28.8	30	31	31.7	32	33.9
**34**	27	29.9	31.2	32	33.2	33.8	34.5
**35**	30	31.5	32	33	33	33.9	34.3
**36**	30	31.5	32.6	33.5	34	34.5	34.88
**37**	30.8	32.1	33	33.7	34.2	34.7	35
**38**	32	33	33.4	34	34.5	35	35.4
**39**	32	33	33.6	34.1	34.7	35.3	35.6
**40**	32.5	33.2	33.8	34.2	34.8	35.4	36
**41**	32.8	33.5	34	34.4	35	35.4	36
**42**	32.8	33.4	34	34.4	34.7	35	35.8

**Table 4:** Percentile values of length (centimeter) for each gestation from 28 to 42 weeks

Gestational age (in weeks)	3^rd^	10^th^	25^th^	50^th^	75^th^	90^th^	97^th^
**28**	34	34	34.9	35.6	37.5	42.3	42.4
**29**	38	38.8	42.8	43.3	45.6	46.5	46.7
**30**	33	35	40.3	44	45.5	45.7	46
**31**	41	41	42.8	43.4	45.7	46.2	46.8
**32**	35.9	39.5	42	44.5	46.1	47.9	48.7
**33**	38.1	42.6	43.5	44	45	46.2	48.2
**34**	42.6	44	45	45.9	46.7	48	49.9
**35**	43.6	45	46	46	47	48	49.3
**36**	43.8	45.5	46	47	48.5	49.6	50.8
**37**	44	45.8	46.8	47.8	49	50.2	51.3
**38**	46	46.9	47.5	49	50.5	51.6	52.4
**39**	46.4	46.9	48	49.1	50.8	51.6	52.5
**40**	46	47	48	49.8	51	51.8	52.8
**41**	46.8	47.3	48.5	50	51	52	53
**42**	46	46.8	48	49.2	50.2	51.2	52.5

**Fig. 2:**
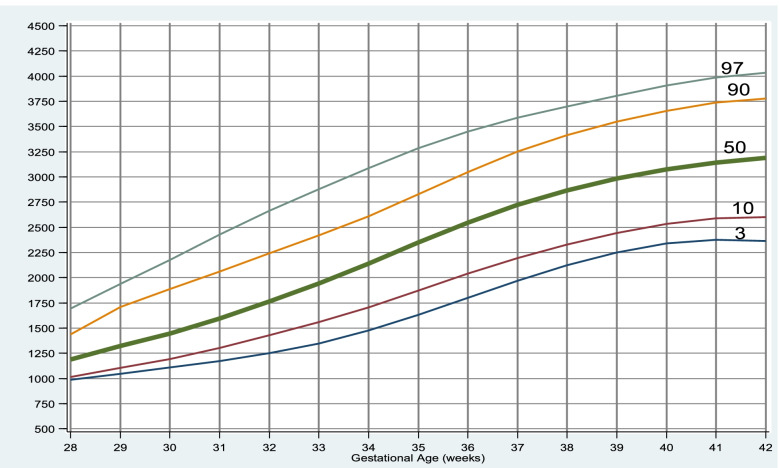
Gestational age specific percentile curve for birth weight

**Fig. 3:**
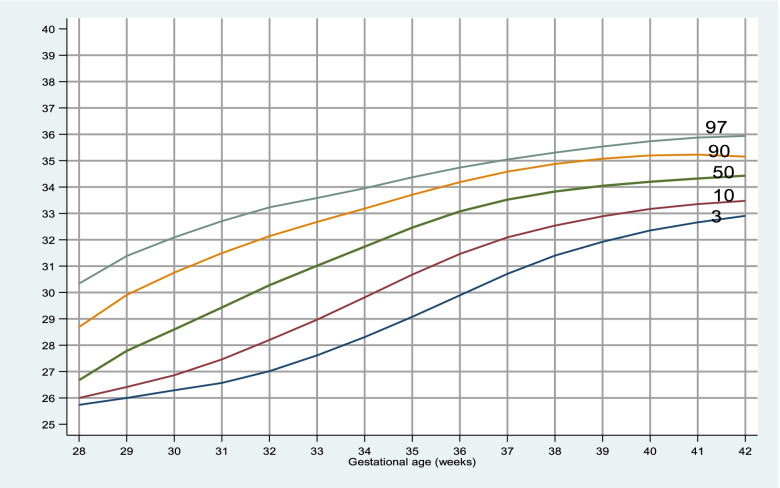
Gestational age specific percentile curve for head circumference

**Fig. 4:**
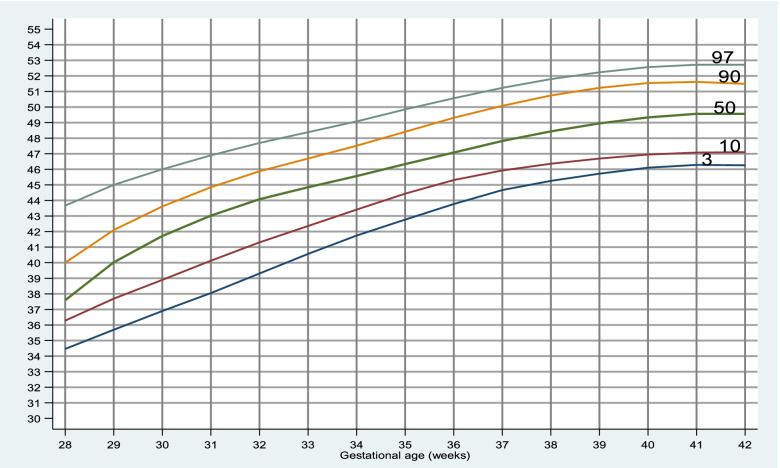
Gestational age specific percentile curve for length

Growth curves are shown in Figs. 2, 3 and 4.

## Discussion

BPKIHS is the tertiary care hospital in Eastern Nepal with highest number of deliveries. It includes mixed group of population that represents different ethnicity, races and castes. Data on birth weight indicates important role of geographic location as an environmental factor on fetal growth. This signifies that regional individualized growth charts for each population is an ideal method for evaluation [9, 10]. Hence, construction of percentile charts for this population prepared according to the features advised by WHO would create ideal reference for the country [11].

A WHO multicenter study reported that the average birth weight of Nepalese newborns was 2780 gms [12]. Our study has shown higher overall mean birth weight (2852.02 gms) as compared to WHO study. The mean birth weight at 40 weeks of gestation was 3123.43gms which was similar to a study done by Manandhar et al. in a tertiary care hospital in Kathmandu who has reported it as 3100 gms [13] and higher than the report of Aryal et al. where mean birth weight of babies at 40 weeks was 3023 gms [3]. This mean birth weight was much higher than that of other countries of this region where the studies from Bangladesh reported it as 2679.0 ± 431.43 to 2,889 ± 468 gms [14, 15] and of India 2666 to 2945 ± 516 gm [16, 17]. As we have not studied maternal factors affecting birth weight like socioeconomic status, consanguinity, gravida status and paternal factors, it would be difficult to explain the difference in mean birth weights between these studies.

The mean head circumference of neonates in our study was 33.6 ± 1.55 cm, with male and female neonates having HC of 33.6 ± 1.67 cm and 33.61 ± 1.38 cm respectively which is lower in overall (36.55 ± 1.189 cm) as well as both male (36.57 ± 4.60 cm) and female (36.54 ± 4.67 cm) compared to study done in Western Rajasthan [10]. The neonates included in the above study included only term neonates from gestational age of 37-42 weeks which could be the cause for higher HC compared to our study. However at 40 weeks of gestation the mean HC in study our study was 34.25 ± 0.87 cm which is similar to Manandhar et al. of 34 ± 1.2 cm but much higher than that by Aryal et al. and Lubchenco et al. which were 33.61 ± 1.52 and 33.8 cm respectively [3, 6, 13]. Similarly, In a study done in British Columbia, the mean head circumference of male 34.70 ± 1.64 cm was higher than that of females 34.13 ± 1.52 cm which was higher in both gender as compared to our study [18].

Birth length at 40 weeks of gestation was found to be similar in our study (49.61 ± 1.85 cm) compared with that of Aryal et al. (49.22 ± 1.52 cm), Lubchenco et al. (49.4) and Manandhar et al. (49.2 ± 2.2 cm) [3, 6, 13]. Similarly, males ( 51.98 ± 2.84 cm) were longer than females ( 51.23 ± 2.72 cm) similar to our study but with lower mean value [18].

In comparison to our data with that of Lubchenco, et al. suggests that the 10th and 90th centiles of our neonates are lower. This resulted in overestimation of the incidence of SGA and underestimation of LGA babies, leading to many AGA neonates labelled as SGA, and LGA neonates being overlooked as they are falsely classified as appropriate for gestational age (AGA). This highlights the importance of population specific and updated growth charts.

The limitations of the study are the gestational age of the neonates were not ultrasonography based but rather mostly based on LMP which may have error due to various factors like educational status of mother leading to under or over estimation of gestational age. The numbers of preterm infants were less in number similar to other studies. Factors other than ethnicity like altitude, maternal size, parity, smoking, parental social position which can affect fetal growth could confound the ethnic differences which has been observed in numerous other studies were not analyzed in detail and lastly it was a single centered study.

## Conclusions

The study concluded that the mean birth weight, head circumference and length of the single live neonates from 28 to 42 weeks of gestation was 2852.02 ± 571.89 gms, 33.6 ± 1.55 cm and 48.42 ± 2.48 cm respectively.

 In this study, we have established local relevant gestational age specific percentile chart and growth curves for Birth weight, Head circumference and length which might be appropriate for babies born in this region.

## Data Availability

The dataset used and/ or analyzed during the current study are available from the corresponding author on reasonable request.

## Abbreviations

AGA: Appropriate for Gestational Age
BPKIHS: Bishweshwar Prasad Koirala Institute of Health Sciences
GDM: Gestational Diabetes Mellitus
HC: Head Circumference.
IRC: Institutional Review Committee
LGA: Large for Gestational Age
LMP: Last Menstrual Period
SD: Standard Deviation
SGA: Small for Gestational Age
USG: Ultrasonography
WHO: World Health Organization
